# Temporal proteomic profiling via 4D-DIA reveals early defense mechanisms and core resistance determinants in soybean against *Phakopsora pachyrhizi*

**DOI:** 10.1007/s44154-025-00268-z

**Published:** 2025-10-27

**Authors:** Zihua Lu, Cong Han, Chao Li, Kelin Deng, Zhihui Shan, Shuilian Chen, Hongli Yang, Yuanxiao Yang, Zhonglu Yang, Hongwei Wang, Haifeng Chen, Qingnan Hao

**Affiliations:** 1 Oil Crops Research Institute, Chinese Academy of Agricultural Sciences, Wuhan, 430062 China; 2 Graduate School, Chinese Academy of Agricultural Sciences, Beijing, 100081 China; 3 Key Laboratory of Biology and Genetics Improvement of Oil Crops, Ministry of Agriculture and Rural Affairs, Wuhan, 430062 China; 4 Department of Plant Genetics and Breeding, College of Agronomy, Shandong Agricultural University, Taian, 271018 China

**Keywords:** Soybean, *Phakopsora pachyrhizi*, 4D-DIA proteomics, Plant immunity, Resistance mechanisms, Molecular breeding

## Abstract

Asian soybean rust, caused by *Phakopsora pachyrhizi*, is a devastating fungal disease threatening global soybean production, particularly in tropical regions where chemical control is increasingly unsustainable. This study employed cutting-edge 4D-DIA proteomics to investigate molecular defense mechanisms in resistant (SX6907) and susceptible (Tianlong 1) soybean cultivars during early infection (12 hpi and 3 dpi). We identified 12,852 proteins, with 1,510 differentially expressed proteins (DEPs) revealing genotype-specific responses. Resistant plants exhibited sustained upregulation of immune receptors (CRKs, LRR-RLKs), MAPK signaling components, and cell wall reinforcement proteins (peroxidases, XTHs), alongside dynamic modulation of calcium signaling and ROS homeostasis. These patterns suggest key pathways enriched in resistance may include phenylpropanoid biosynthesis, isoflavonoid production, and ER stress responses, while susceptible plants showed suppression of photosynthesis and defense pathways. Weighted Protein Co-expression Network Analysis(WPCNA) highlighted co-expression modules linked to resistance, potentially including NLR-mediated effector-triggered immunity. Crucially, DIR proteins and organelle-specific defense hubs (e.g., chloroplasts, nuclei) were implicated in rust resistance. Validation by qPCR confirmed concordance for 84% of tested DEPs. Our findings provide a protein-level blueprint of soybean rust resistance, identifying candidate targets for marker-assisted breeding and genetic engineering to develop durable resistant varieties, reducing reliance on fungicides.

**Supplementary Information:**

The online version contains supplementary material available at 10.1007/s44154-025-00268-z.

## Introduction

Soybean (*Glycine max *(L.) Merr.) is one of the most economically significant legume crops worldwide, serving as a primary source of plant-based protein and oil for human consumption, animal feed, and industrial applications (https://www.fao.org/statistics/en/). The global production of soybean exceeds 350 million metric tons annually, with major contributions from the United States, Brazil, Argentina, and China (https://www.nass.usda.gov/). However, soybean cultivation faces persistent challenges from biotic stresses, among which fungal diseases pose severe threats to yield and quality.

Asian soybean rust, caused by the obligate biotrophic fungus *Phakopsora pachyrhizi*, is considered one of the most devastating foliar diseases of soybean (Goellner et al. 2010). First reported in Japan in 1902, the pathogen has since spread across Asia, Africa, Australia, and the Americas, causing yield losses ranging from 10 to 80% depending on environmental conditions and cultivar susceptibility (Yorinori et al. 2005). Unlike other rust pathogens, *Phakopsora pachyrhizi* exhibits a broad host range, infecting over 150 legume species, which complicates disease management strategies (Favoretto et al. 2025).

With the global soybean production center shifting to South America, the region has become the world's largest soybean producer, with a total output of 210 million tons in 2023 and an average seasonal production value of $115 billion (Gupta et al. 2023). However, the tropical and subtropical climates of South America provide ideal conditions for soybean rust, making the disease ubiquitous in soybean-growing areas. In Brazil alone, the cost of controlling soybean rust exceeds $2 billion per season (Godoy et al. 2015), with 60% of pesticides used annually targeting this disease. Over the past three years, pesticide usage has far surpassed the total amount used in the previous decade (http://cn.agropages.com/), nearing its limit. Moreover, the environmental damage caused by pesticide application and the growing issue of pathogen resistance (Godoy 2011; Klosowski et al. 2016), have led to a critical situation where soybean rust may soon become "untreatable" (Godoy et al. 2016; Juliatti et al. 2025). These challenges underscore the critical need for alternative control measures, particularly the development of genetically resistant soybean cultivars through molecular breeding and biotechnological approaches.

Plants have evolved a sophisticated two-layered immune system to combat pathogen invasion, consisting of pattern-triggered immunity (PTI) and effector-triggered immunity (ETI) (Jones and Dangl 2006). The primary defense barrier, PTI, is initiated when plant pattern recognition receptors (PRRs) identify conserved pathogen-associated molecular patterns (PAMPs), such as fungal-derived chitin (Miya et al. 2007). In soybean, this recognition process primarily involves receptor-like kinases (RLKs) and receptor-like proteins (RLPs) that specifically detect *Phytophthora pachyrhizi* and trigger subsequent defense signaling (Zhang et al. 2024). Key early PTI responses include: (1) rapid calcium influx and reactive oxygen species (ROS) production (Torres et al. 2006); (2) activation of mitogen-activated protein kinase (MAPK) signaling pathways (Zhang and Klessig 2001); and (3) callose deposition at infection sites to fortify cell walls (Luna et al. 2011).

If pathogens overcome PTI by secreting effector proteins that suppress host defenses, plants deploy ETI, typically mediated by nucleotide-binding leucine-rich repeat (NLR) proteins (Monteiro and Nishimura 2018). In soybean, several resistance (R) locus (*Rpp1*-*Rpp7*, *Rpp6907*) have been identified, conferring partial or complete resistance to *Phakopsora pachyrhizi *(Chen et al. 2015; Chicowski et al. 2024; Childs et al. 2018). ETI typically induces a hypersensitive response (HR), characterized by localized programmed cell death to contain pathogen proliferation, and systemic acquired resistance (SAR) mediated by salicylic acid signaling (Vlot et al. 2009).

Complementing these immune responses, soybean synthesizes an array of antimicrobial compounds, including: (1)isoflavonoids such as glyceollins, which function as potent phytoalexins with broad-spectrum antifungal activity (Sohn et al. 2021); and (2) phenylpropanoid pathway metabolites that contribute to cell wall reinforcement through lignin deposition (Dixon et al. 2002). Despite this multi-faceted defense system, *Phakopsora pachyrhizi* has evolved sophisticated strategies to circumvent host immunity, highlighting the need for more comprehensive molecular characterization of soybean-pathogen interactions to develop effective disease management strategies.

Proteomics, the large-scale study of proteins, plays a pivotal role in elucidating post-translational modifications (PTMs), protein–protein interactions, and dynamic changes in protein abundance under stress conditions (Dixon et al. 2002). Unlike transcriptomics, which only assesses mRNA levels, proteomics directly measures functional protein expression, providing a more accurate representation of cellular responses. Early proteomic techniques, such as two-dimensional gel electrophoresis (2D-GE) coupled with matrix-assisted laser desorption/ionization time-of-flight (MALDI-TOF) mass spectrometry, were limited by low sensitivity and poor reproducibility (Görg et al. 2004). However, the introduction of liquid chromatography-tandem mass spectrometry (LC–MS/MS) marked a significant breakthrough, enabling high-throughput protein identification and quantification (Shuken 2023).

Modern proteomics employs two primary mass spectrometry acquisition methods: data-dependent acquisition (DDA, or shotgun proteomics) and data-independent acquisition (DIA, e.g., SWATH-MS). DDA selects the most intense peptide ions for fragmentation, often resulting in stochastic data gaps and reduced reproducibility (Michalski et al. 2011). In contrast, DIA systematically fragments all peptides within predefined mass windows, significantly improving quantitative accuracy and experimental reproducibility (Gillet et al. 2012). These advancements have greatly enhanced the depth and reliability of proteomic analyses.

While transcriptomic and metabolomic approaches have significantly advanced our understanding of soybean rust resistance (Hossain et al. 2018; Panthee et al. 2009), a critical knowledge gap remains in the proteomic dimension, particularly during the crucial early infection phases. This study leverages cutting-edge 4D-DIA("4D" refers to the addition of a fourth dimension—ion mobility separation—to the traditional three dimensions of liquid chromatography, precursor mass-to-charge (m/z), and fragment m/z, significantly enhancing resolution and sensitivity.) proteomics to comprehensively characterize the soybean defense proteome, with four key objectives: (1) systematic identification of defense-related proteins including pathogenesis-related (PR) proteins, RLKs, and NLRs; (2) quantitative profiling of temporal protein abundance changes following *Phakopsora pachyrhizi* infection; (3) elucidation of post-translational modifications (PTMs) that modulate immune signaling cascades; and (4) comparative analysis between resistant and susceptible cultivars to identify robust molecular markers of resistance.

This study aims to employ 4D-DIA quantitative proteomics to dissect soybean defense mechanisms against *Phakopsora pachyrhizi*, providing a comprehensive protein-level understanding of resistance traits. By integrating proteomics with existing transcriptomic and metabolomic data, we seek to map the soybean proteome dynamics during rust infection;Identify candidate resistance proteins for molecular breeding; Propose novel strategies for durable rust resistance.The findings will contribute to global efforts in developing next-generation rust-resistant soybean varieties, ensuring sustainable crop production in the face of evolving pathogen threats.

## Results

### Plant materials, pathogen cultivation and inoculation

**Fig. 1:**
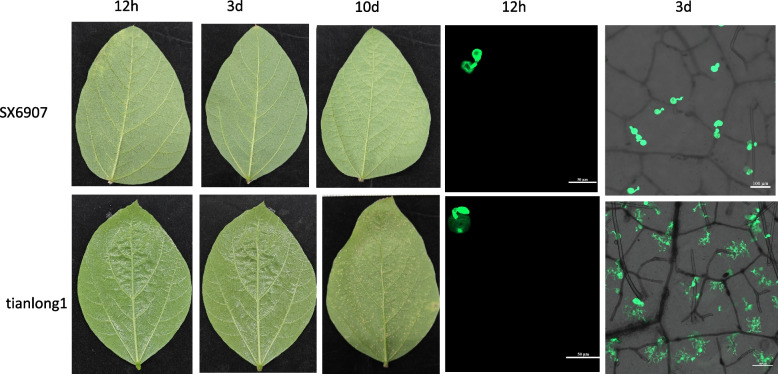
Asian soybean rust resistance phenotypes, microscopic observation after rust SS4 infection

The soybean germplasm SX6907 exhibits strong resistance and demonstrates an immune response to *Phakopsora pachyrhizi* isolate SS4 (Shan et al. 2012). In contrast, the high-yielding cultivar Tianlong 1, bred by the Oil Crops Research Institute of the Chinese Academy of Agricultural Sciences (OCRI), is susceptible to this pathogen. For inoculation experiments, soybean seeds were grown in a greenhouse under controlled conditions (24–26 °C, 18/6 h light/dark cycle). At 14 days post-cultivation, fully expanded primary leaves were collected and inoculated with *Phakopsora pachyrhizi* SS4. Leaf samples were then harvested at two time points post-infection: 12 h (hpi) and 3 days (dpi). The phenotypic and microscopic observations of resistant and susceptible materials after inoculation with rust fungus are shown in Fig. 1. The selection of 12 hpi and 3 dpi as critical time points is based on the following rationale: 12 hpi captures the initial fungal invasion stage, including appressorium differentiation, penetration, and primary hyphae formation, allowing the study of early plant defense signaling before visible symptoms (Ouyang et al. 2024); whereas 3 dpi represents the establishment of the parasitic relationship with haustoria formation and the plant's full-scale defense response, characterized by a clear divergence between susceptible (e.g., cell deformation) and resistant (e.g., hypersensitive response) interactions, reflecting the critical battle between effector-triggered immunity and susceptibility. Additionally, non-inoculated control samples were collected at corresponding time points (hpu/dpu). The study included eight experimental groups: Tianlong 1 inoculated at 12 hpi (S-12 hpi) and 3 dpi (S-3 dpi); SX6907 inoculated at 12 hpi (R-12 hpi) and 3 dpi (R-3 dpi); Tianlong 1 non-inoculated controls at 12 hpu (S-12 hpu) and 3 dpu (S-3 dpu); and SX6907 non-inoculated controls at 12 hpu (R-12 hpu) and 3 dpu (R-3 dpu), with three biological replicates collected for each group.

The inoculation procedure was performed according to the method described by Chen et al. (2015). *Phakopsora pachyrhizi* urediniospores were propagated on susceptible Tianlong 1 leaves, collected, and suspended in 0.01% (v/v) Tween 20 solution at a concentration of 1 × 10^5^ spores/mL. Detached soybean leaves were placed on moist filter paper in Petri dishes, with their adaxial surfaces covered by an additional filter paper layer. Each plate contained 5–6 leaves, and each leaf was inoculated with four 5μL droplets of the spore suspension. Following inoculation, the plates were incubated in a growth chamber at 24℃ and 70% relative humidity under a 12/12 h light/dark photoperiod. To maintain humidity, 1–2 mL of sterile water was added daily. Leaf samples were collected at 12 h post-inoculation (hpi) and 3 days post-inoculation (dpi), with three biological replicates per time point.

### Protein quality assessment

**Fig. 2:**
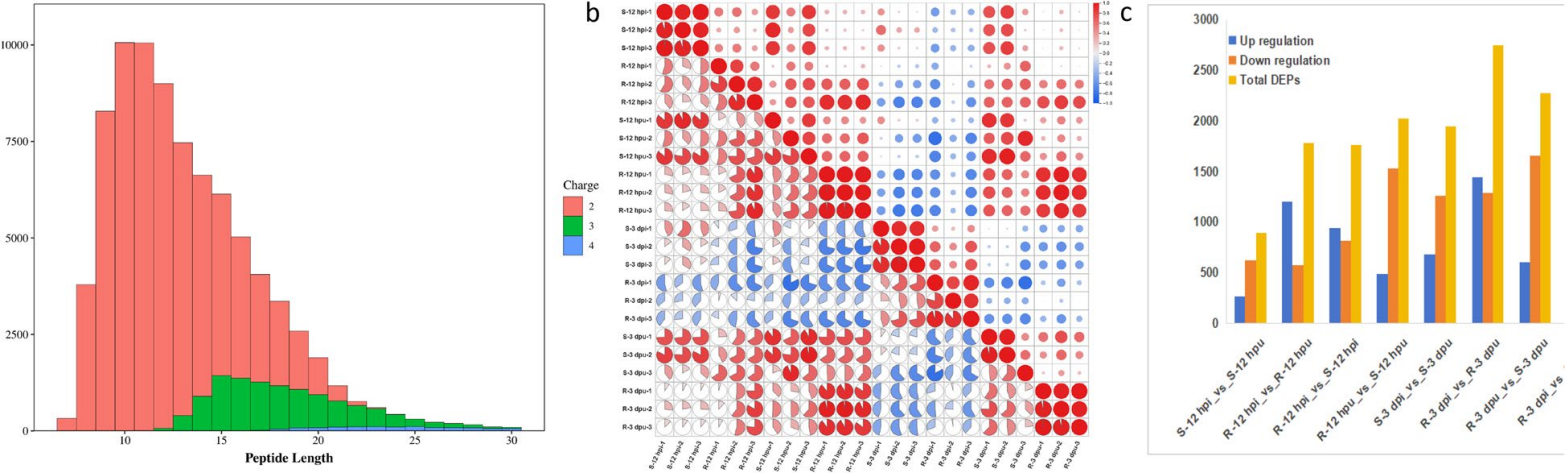
Protein quality assessment (**a**) Peptide length distribution.The x-axis represents peptide length, the y-axis represents the number of peptides corresponding to each length, and different colors indicate the detected charge states of the peptides. **b** Inter-sample Correlation Plot. The x-axis and y-axis represent sample names, and the color gradient from red to yellow indicates the correlation strength from high to low. **c** Display of differentially expressed proteins in the eight comparison groups

Following mass spectrometry data analysis, comprehensive quality control assessments—including peptide length distribution, peptide count distribution, and missed cleavage site analysis—were performed to ensure data reliability. A total of 77,568 peptides and 12,852 proteins were identified across all samples. Specifically, protein detection yielded 11,970 (S-12 hpi), 11,897 (R-12 hpi), 12,080 (S-12 hpu), 11,552 (R-12 hpu), 12,057 (S-3 dpi), 11,453 (R-3 dpi), 12,152 (S-3 dpu), and 11,553 (R-3 dpu) proteins, respectively.The majority of peptides (7–20 amino acids in length) conformed to the expected range for tryptic digestion and MS/MS fragmentation, meeting established quality control criteria (Fig. 2a). Inter-sample correlation analysis further validated the reproducibility of biological replicates, with stronger within-group correlations than between-group correlations, reinforcing the reliability of subsequent differential expression analyses. Replicate samples demonstrated high consistency, fulfilling all quality assurance benchmarks (Fig. 2b).

**Fig. 3:**
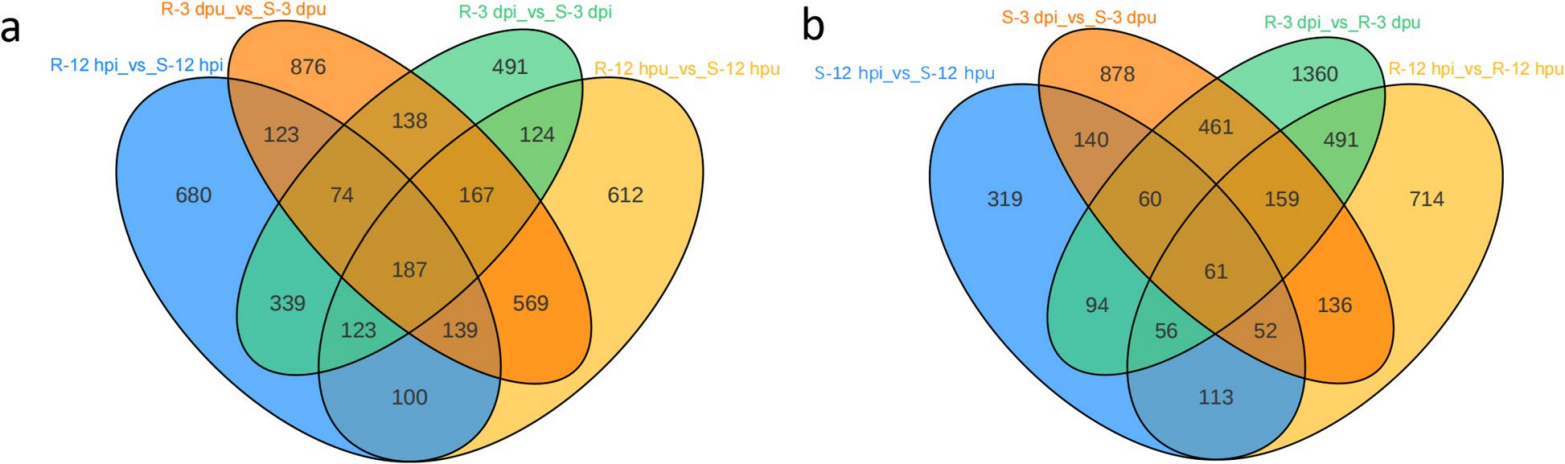
The differentially expressed protein (DEP) profiles. **a** Venn diagram of DEPs in resistant vs. susceptible comparisons. **b** Venn diagram of DEPs in inoculated vs. non-inoculated comparisons

In this study, significantly differential proteins were screened using the following criteria: for two-group comparisons, proteins with a fold change (FC) ≥ 1.5 or ≤ 0.6667 and a P-value ≤ 0.05 were considered significant, while for multi-group comparisons (≥ 3 groups), only a P-value ≤ 0.05 was required. Across all analyses, we identified 15,068 differentially expressed proteins (DEPs), comprising 6,296 upregulated and 8,772 downregulated proteins in the eight-group comparison (Fig. 2c). Further analysis revealed that rust infection triggered 1,510 DEPs in resistant versus susceptible comparisons, including 339 common DEPs shared between both infection time points (Fig. 3a). Notably, comparative profiling demonstrated distinct response patterns: 2,565 proteins were exclusively regulated in resistant materials, 1,337 proteins showed specific responsiveness in susceptible materials, and 1,192 proteins exhibited conserved differential expression in both material types upon infection (Fig. 3b).

### GO analysis of DEPs

This study aimed to identify significantly enriched GO terms and characterize the key biological pathways activated during fungal infection. Comparative proteome profiling revealed that "response to stimulus" was the most prominently enriched functional category in both cultivars following pathogen challenge(Table S1). Notably, resistant plants exhibited 2.3-fold more DEPs than susceptible plants, demonstrating their enhanced capacity for immune perception and signaling.

**Fig. 4:**
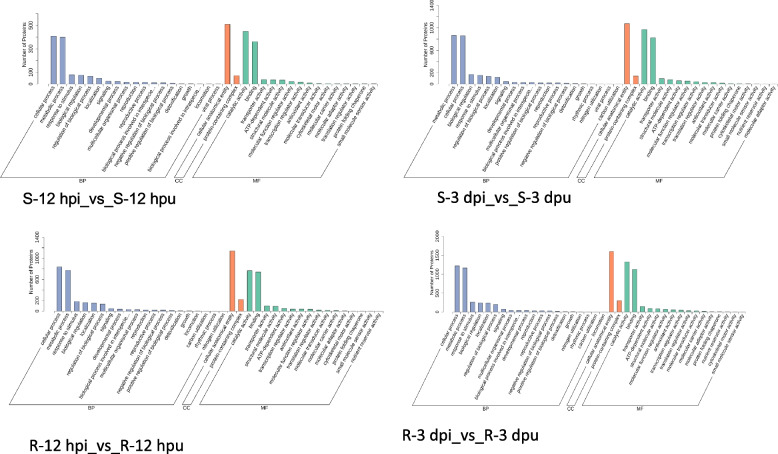
Gene Ontology (GO) enrichment analysis of the DEPs

Time-course analysis revealed distinct defense activation patterns between genotypes. Resistant plants maintained remarkably stable levels of stimulus-responsive proteins (163 at 12 hpi vs 160 at 3 dpi), indicating sustained defense engagement throughout infection (Fig. 4). In contrast, susceptible plants showed only transient induction, with defense-related transcripts rapidly declining after initial detection.

Detailed analysis of 14 specific GO terms uncovered genotype-specific responses in key biological processes(Table S2):

Cell wall processes (GO:0071555, GO:0009833, GO:0009834, GO:0042545):At 12 hpi, susceptible plants showed predominant downregulation in cell wall organization (9 down vs 2 up), while resistant plants exhibited strong upregulation (15 up vs 3 down), suggesting active cell wall reinforcement. By 3 dpi, susceptible plants displayed marked downregulation of cell wall-related genes, whereas resistant plants maintained upregulated expression.

For defense response (GO:0006952, GO:0098542, GO:0051707), resistant plants at 12 hpi showed significant upregulation (15 up vs. 9 down), indicating early defense activation. In contrast, susceptible plants at 3 dpi had a mixed response (5 up, 9 down), suggesting a delayed or weaker defense. Resistant plants at 3 dpi continued to exhibit sustained upregulation (19 up vs. 13 down), reinforcing their resistance. In the response to oxidative stress (GO:0006979), resistant plants at 12 hpi showed strong downregulation (4 up vs. 24 down), possibly due to better control of reactive oxygen species (ROS). This trend intensified by 3 dpi (4 up vs. 32 down), suggesting ROS homeostasis is effectively maintained in resistant plants.

For pectin catabolism (GO:0045490), susceptible plants at 3 dpi displayed strong downregulation (1 up vs. 11 down), possibly linked to cell wall weakening. Resistant plants followed a similar but less pronounced trend (1 up vs. 9 down).

In the response to symbiotic fungus (GO:0009610), resistant plants at 12 hpi had more downregulated genes (1 up vs. 6 down), possibly suppressing symbiosis-related pathways. By 3 dpi, both susceptible and resistant plants showed downregulation, suggesting a shift toward defense rather than symbiosis.

Resistant plants demonstrate early and sustained upregulation of defense-related genes, particularly in cell wall reinforcement and defense responses. In contrast, susceptible plants exhibit downregulation of cell wall organization genes, potentially weakening structural defenses. Oxidative stress management differs significantly, with resistant plants more effectively suppressing ROS-related genes. Additionally, pectin catabolism is downregulated upon infection, possibly affecting cell wall integrity. These findings highlight key molecular differences between resistant and susceptible plant responses to infection.

### Functional characterization of DEPs through KOG annotation

**Fig. 5:**
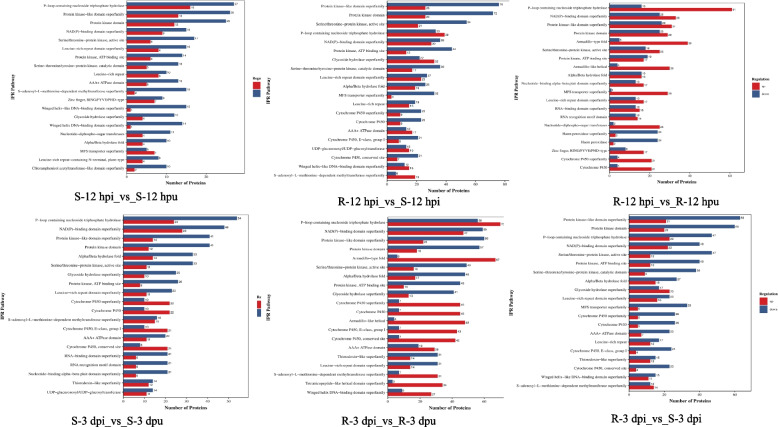
Clusters of KOG functional annotation

To systematically elucidate the functional divergence between rust-resistant and susceptible soybean genotypes, we performed extensive eukaryotic KOG classification of DEPs. The KOG annotation revealed that the 1,243 identified DEPs were successfully classified into 20 functionally distinct categories (Fig. 5). The resistant group (R) exhibited a substantially higher number of DEPs compared to the susceptible group (S), particularly in the 12-h and 3-day comparisons. Notably, defense-related genes (V) and nuclear structure-associated genes (Y) were specifically activated in the resistant group, while cell motility (N) showed minimal changes.

Functional categorization showed highest DEP counts in general function prediction (R), followed by energy metabolism (C) and signal transduction (T). Notably, the R group displayed unique upregulation of nuclear structure (Y) and defense-related genes (V), while these pathways remained unchanged in the S group. Temporal analysis demonstrated increased transcriptional responses at 3 days post-infection compared to 12 h. The consistent activation of defense mechanisms (V) in the R group suggests their potential role in disease resistance, and the marked upregulation of uncharacterized proteins (S) in resistant plants highlights important knowledge gaps requiring further investigation. These findings provide valuable insights into potential molecular mechanisms underlying pathogen resistance.

### KEGG pathway of DEPs

To elucidate the biological pathways associated with DEPs, KEGG pathway enrichment analysis was conducted. In the S-12 hpi vs. S-12 hpu comparison, 423 DEPs were mapped to 121 KEGG pathways, whereas the R-12 hpi vs. R-12 hpu comparison identified 1261 DEPs linked to 131 pathways. Similarly, at 3 dpi, 1716 DEPs in S-3 dpi vs. S-3 dpu and 2034 DEPs in R-3 dpi vs. R-3 dpu were annotated to 133 and 134 pathways, respectively. These results indicate that rust infection induces extensive reprogramming of multiple biological pathways, with distinct patterns between R and S genotypes across different infection stages.

**Fig. 6:**
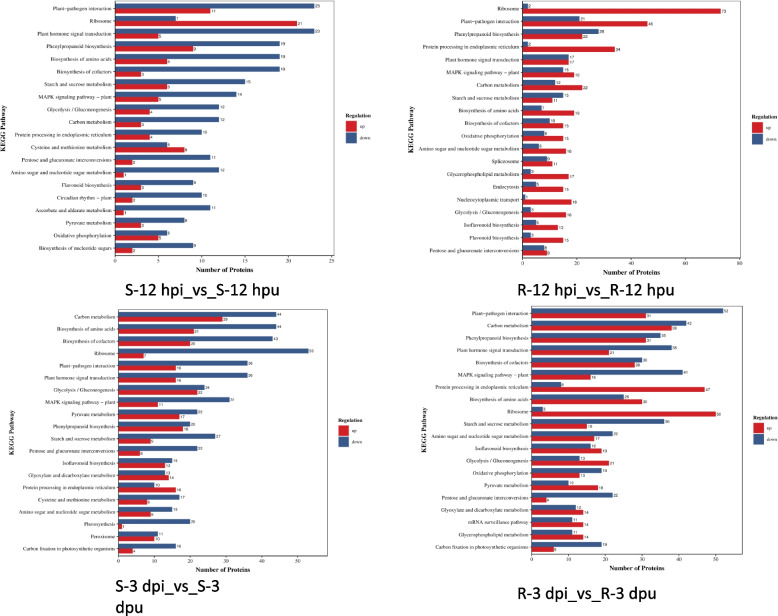
KEGG enrichment analysis of DEPs

Notably, resistant plants exhibited stronger activation of defense-related pathways following rust infection (Fig. 6)(Table S2). The plant-pathogen interaction pathway (ko04626) contained the highest number of DEPs, particularly in R samples (67 DEPs at 12 hpi; 83 at 3 dpi), suggesting an active defense response. Additionally, MAPK signaling (ko04016) and plant hormone signal transduction (ko04075) were more upregulated in R samples, indicative of robust stress signaling. Flavonoid (ko00941) and isoflavonoid biosynthesis (ko00943) pathways were significantly induced in R plants, likely contributing to antimicrobial defense.

Resistant plants displayed enhanced endoplasmic reticulum (ER) stress responses and protein processing. The protein processing pathway in the ER (ko04141) was strongly upregulated in R samples (36 DEPs at 12 hpi; 55 at 3 dpi), implying a role for ER stress in defense. Furthermore, the phagosome pathway (ko04145) was uniquely upregulated in R samples (13 DEPs at 12 hpi; 22 at 3 dpi), potentially facilitating pathogen degradation. In contrast, susceptible plants showed significant downregulation of photosynthesis-related pathways (ko00195, ko00196) at 3 dpi (21 and 1 DEPs, respectively), reflecting metabolic disruption due to infection. Resistant plants, however, maintained partial photosynthetic activity, suggesting better stress tolerance.

Phenylpropanoid biosynthesis (ko00940) was highly active in both genotypes but exhibited stronger upregulation in R plants (66 DEPs at 3 dpi). This pathway likely contributes to lignin and flavonoid production, reinforcing cell walls and enhancing defense. Autophagy (ko04136) was slightly induced in R-3 dpi (4 upregulated genes), possibly aiding in cellular clearance during defense. Peroxisome function (ko04146) was predominantly downregulated in S samples but displayed mixed regulation in R plants, potentially reflecting reactive oxygen species (ROS) management strategies.

The substantial enrichment of DEPs in these key pathways underscores their critical roles in rust resistance and susceptibility. These findings provide valuable insights for future genetic improvement strategies aimed at enhancing disease resistance in crops.

### Subcellular location and protein domain analysis of DEPs

**Fig. 7:**
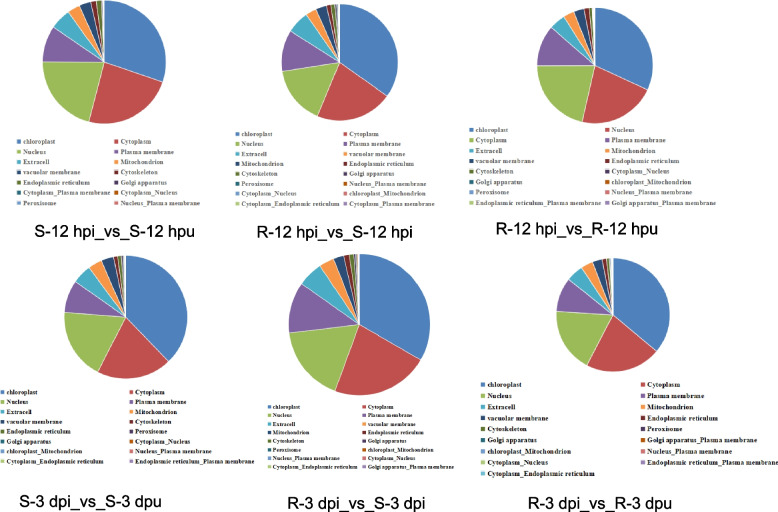
Subcellular locations analysis of differentially abundant proteins

Protein subcellular localization serves as a crucial dynamic regulatory mechanism that governs protein function, interaction networks, and ultimately cellular homeostasis. Our quantitative analysis of DEPs revealed striking compartment-specific distribution patterns that delineate key defense strategies in resistant versus susceptible plants (Fig. 7).

Chloroplasts emerged as the primary defense hub, showing the most pronounced DEP counts across all comparisons. Resistant genotypes maintained robust upregulation at both timepoints (R-12hpi:383 up/186 down; R-3dpi: 426 up/563 down), while susceptible plants exhibited progressive downregulation (S-12hpi: 86 up/185 down; S-3dpi: 181 up/555 down). This compartment-specific pattern suggests chloroplasts serve dual roles as early signaling centers through retrograde signaling pathways and as sustained defense factories for producing secondary metabolites and reactive oxygen species. The dramatic downregulation in susceptible plants likely reflects pathogen strategies to disable photosynthetic defense mechanisms.

Membrane systems displayed polarized responses, with plasma membrane-localized proteins showing one of the most divergent patterns between genotypes. Resistant samples demonstrated remarkable upregulation (R-12hpi: 174 up/30 down; R-3dpi: 191 up/76 down), representing enhanced pathogen recognition through receptor kinases, increased transporter activity for defense compound secretion, and active cell wall remodeling. In contrast, susceptible plants showed modest changes with downregulation bias (S-12hpi: 29 up/56 down), suggesting suppression of membrane-based surveillance systems.

Nuclear-cytoplasmic coordination was particularly evident in resistant plants, with sustained upregulation in both compartments (R-12hpi nucleus: 249 up/137 down; cytoplasm: 278 up/103 down). This coordinated regulation indicates active transcriptional reprogramming, increased ribosome biogenesis for defense protein production, and potential nucleocytoplasmic trafficking of defense regulators. The more balanced changes observed in susceptible plants may reflect failed attempts to mount effective defenses.

Temporal analysis revealed distinct phases of compartment specialization. Early responses (12hpi) in resistant plants featured pronounced chloroplast and nuclear activation, while later stages (3dpi) showed maintained chloroplast activity with increased vacuolar membrane involvement (R-3dpi: 27 up/50 down). This progression suggests an initial phase of signaling and transcriptional activation followed by deployment of antimicrobial compounds to vacuoles and apoplast. Susceptible plants showed progressive collapse of chloroplast function accompanied by increasing extracellular suppression (S-3dpi extracellular: 32 up/66 down), potentially facilitating pathogen nutrient acquisition.

Secondary compartments displayed specialized roles in the defense response. Peroxisomes showed exclusive upregulation in S-3dpi (6 up/3 down), possibly indicating oxidative stress responses. Chloroplast-mitochondrion dual-localized genes exhibited resistant-specific activation (R-12hpi: 2 up/0 down), suggesting important organelle cross-talk during defense. The cytoskeleton showed minimal overall changes, though susceptible plants displayed consistent downregulation trends.

**Fig. 8:**
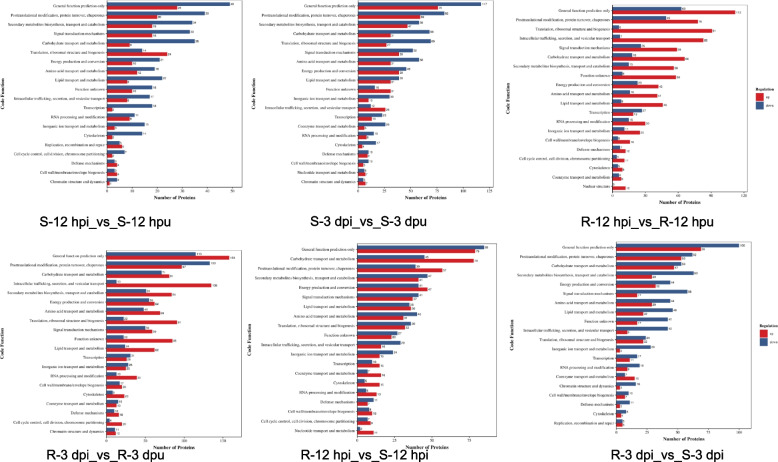
Protein domain analysis of differentially abundant proteins

Our comprehensive domain analysis through InterProScan (v92.0) identified four particularly abundant domain groups among the top 20 most prevalent domains: P-loop containing nucleoside triphosphate hydrolases (IPR027417), protein kinase-like domain superfamily (IPR011009), leucine-rich repeat domains (IPR032675), and cytochrome P450 superfamily (IPR036396), with each category represented by six DEPs (Fig. 8). These domain families play well-documented roles in plant defense, including pathogen recognition (LRR domains), signal transduction (kinase domains), energy metabolism (P-loop NTPases), and secondary metabolite biosynthesis (P450 enzymes), providing valuable insights into the functional specialization of defense-related proteins.

### Visualization of hub protein co-expression networks in defense responses induced by *Phakopsora pachyrhizi* infection

**Fig. 9:**
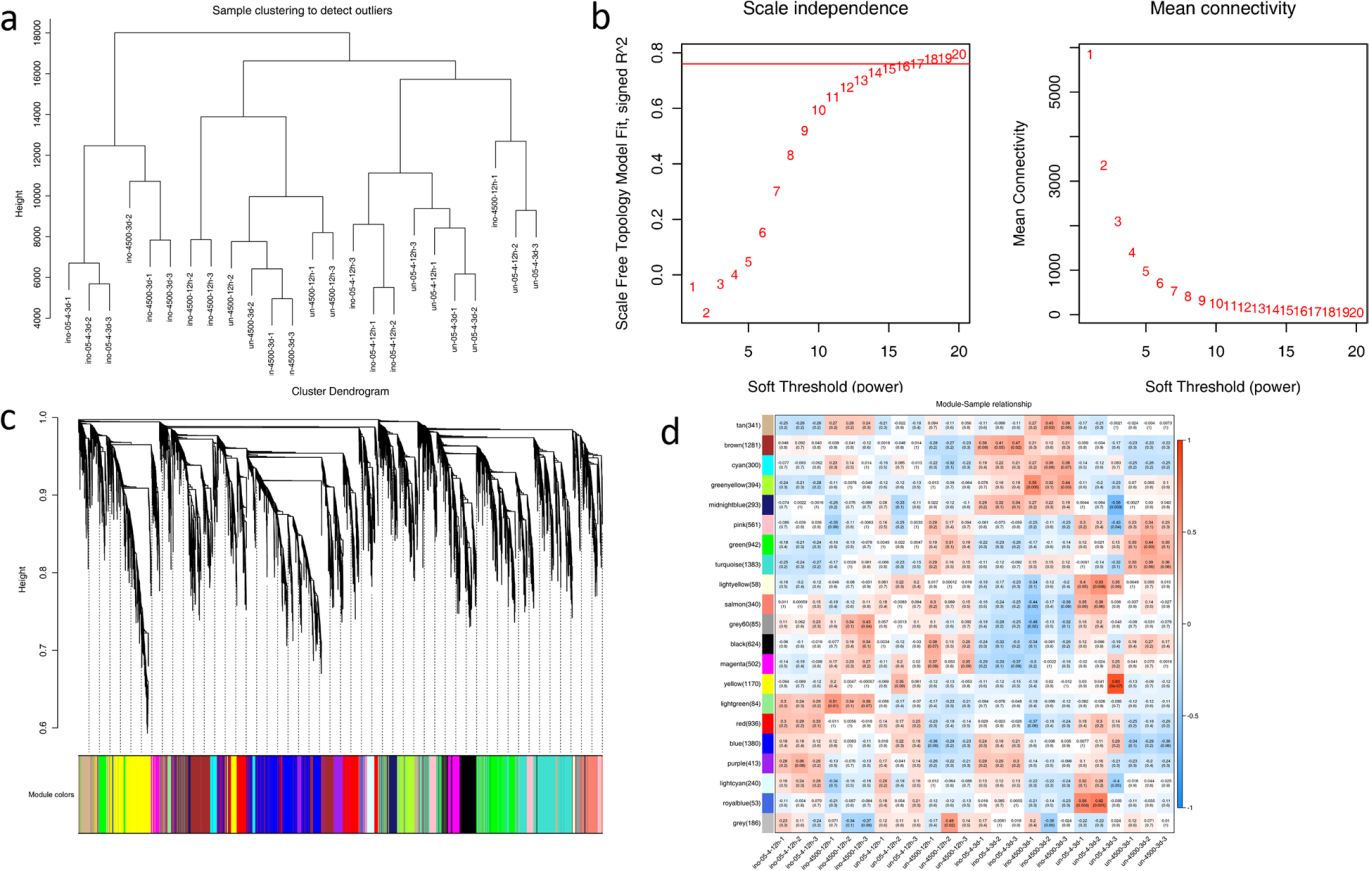
Weighted gene co-expression network analysis of DEPs. **a** Sample clustering to detect outliers; **b** R2 values and average connectivity corresponding to different soft thresholds; **c** Clustering dendrograms of protein and module division, with dissimilarity based on the topological overlap, together with assigned module colours. Overall, 20 co-expression modules were constructed and are shown in different colours. These modules ranged from large to small according to the number of proteins included. **d** Module-sample group association analysis. Each row corresponds to a module, labelled with colour as in panel

WPCNA is a powerful systems biology approach for identifying highly correlated gene/protein modules and detecting hub molecules in biological networks. To elucidate the dynamic proteomic changes during P. pachyrhizi infection and pinpoint critical hub proteins governing defense responses, we analyzed 11,566 high-confidence proteins using the WPCNA package (v1.72). All samples passed rigorous quality control (Fig. 9a), with no outliers detected, ensuring data reliability for downstream analyses. Through scale-free topology criterion evaluation, we determined an optimal soft threshold power of 16 (Fig. 9b), achieving a scale-free fit index (R^2^ > 0.8) while maintaining moderate mean connectivity. This parameter selection facilitated the construction of biologically relevant co-expression networks optimized for identifying key regulatory proteins. The analysis ultimately generated 20 distinct co-expression modules (Fig. 9c), each represented by unique color codes. Notably, the grey module comprised unassigned proteins that failed to meet the stringent correlation thresholds (Pearson’s r > 0.85), which were excluded from further functional analyses.

Module-trait relationship analysis revealed striking associations between specific co-expression modules and infection stages (Fig. 9d). The purple module (413 proteins) showed strong positive correlation with early infection phases in susceptible cultivar SX6907, while the tan module (341 proteins) was uniquely associated with the resistant variety Tianlong1. Functional annotation of the purple module highlighted enrichment in defense-related processes: biological processes (BP) included defense response (GO:0006952), protein phosphorylation (GO:0006468), and carbohydrate metabolism (GO:0005975); molecular functions (MF) featured transmembrane transporter (GO:0022857) and kinase activities (GO:0004674); cellular components (CC) were dominated by membrane systems (GO:0016021). KEGG pathway analysis further implicated this module in plant-pathogen interactions (ko04626) and MAPK signaling (ko04016). Conversely, the tan module in the resistant genotype exhibited distinct enrichment patterns: BP terms included protein folding (GO:0006457) and translation (GO:0006412); MF categories highlighted oxidoreductase (GO:0016702) and RNA-binding activities (GO:0003723); CC analysis revealed endoplasmic reticulum-associated protein processing machinery. KEGG mapping associated this module with isoflavonoid biosynthesis (ko00943) and endoplasmic reticulum protein processing (ko04141), suggesting a specialized defense mechanism in resistant plants. Complete pathway analyses are provided in Supplementary Tables S1–S2.

### Validation of differentially accumulated proteins by qPCR

We evaluated the correlation between mRNA and protein levels using RT-qPCR. We randomly selected 32 differentially expressed proteins for RT-qPCR analysis, All primer sequences are provided in Supplementary Table S4. The results showed that 27 genes exhibited consistent expression patterns at both the protein and mRNA levels. However, discrepancies were observed between the transcriptional and protein levels for 5 genes, which may arise from post-transcriptional and post-translational regulatory processes (Table 1).

**Table 1:** Verification of some differentially accumulated proteins by qPCR

Comparison group	Gene ID	Protein ID	Ratio in iTRAQ	Ratio in qRT PCR
R-12 hpi_vs_R-12 hpu	06G258500	A0A0R0JLC9	3.90	4.46
	19G094100	A0A0R4J614	2.96	6.84
	19G182300	I1NA96	9.63	3.50
R-12 hpi_vs_S-12 hpi	01G127200	I1J7J5	0.31	0.12
	03G047000	A0A0R0KET2	0.72	0.56
	11G176500	A0A0R0HT22	0.88	0.45
	13G193700	A0A0R0GZ65	0.53	0.55
R-12 hpu_vs_S-12 hpu	12G097500	I1LRP3	0.61	0.17
	12G166200	A0A368UHI2	1.63	0.25
	15G136800	I1MGB0	18.03	0.45
R-3 dpi_vs_R-3 dpu	06G162300	I1KBU7	2.05	1.29
	14G085600	A0A0R0GB30	18.20	2.45
	15G089000	C6T9R1	4.65	1.23
	16G043900	I1ML54	3.12	1.13
	18G061100	K7MQ84	6.19	7.99
R-3 dpi_vs_S-3 dpi	09G277800	I1L738	1.55	0.09
	15G232800	A0A0R0GDP3	0.67	0.54
	15G238200	A0A0R0GF16	1.78	1.47
	16G043900	I1ML54	0.46	0.16
R-3 dpu_vs_S-3 dpu	06G040400	K7KT00	1.85	11.33
	15G021400	I1MCW6	1.87	12.71
	16G129300	A0A0R0FQ38	5.18	2.39
	19G242200	I1NC65	1.63	4.42
S-12 hpi_vs_S-12 hpu	06G027700	C6TDZ7	0.67	1.01
	08G107700	A0A0R0IK45	0.54	0.36
	10G058200	I1L905	2.30	1.31
	15G136800	I1MGB0	5.02	0.83
S-3 dpi_vs_S-3 dpu	07G206800	A0A0R0J5W4	0.65	0.44
	08G235400	I1KW54	5.44	63.46
	15G107900	I1MFH3	0.53	0.58
	15G211500	I1MI59	14.09	4.32
	17G128000	I1MUM7	50.06	16.74

## Discussion

### DEPs involved in calcium (Ca^2^⁺) signaling

PTI is activated when PAMPs or DAMPs are recognized by PRRs on the plant cell surface. This recognition triggers a cascade of downstream signaling events leading to defense responses. Ca^2^⁺ signaling is one of the earliest and most critical events in PTI. Upon pathogen recognition, a rapid Ca^2^⁺ influx occurs into the cytosol, triggering downstream defense responses. This process involves calcium sensors, channels, and kinases, which amplify and transduce the immune signal.

**Fig. 10:**
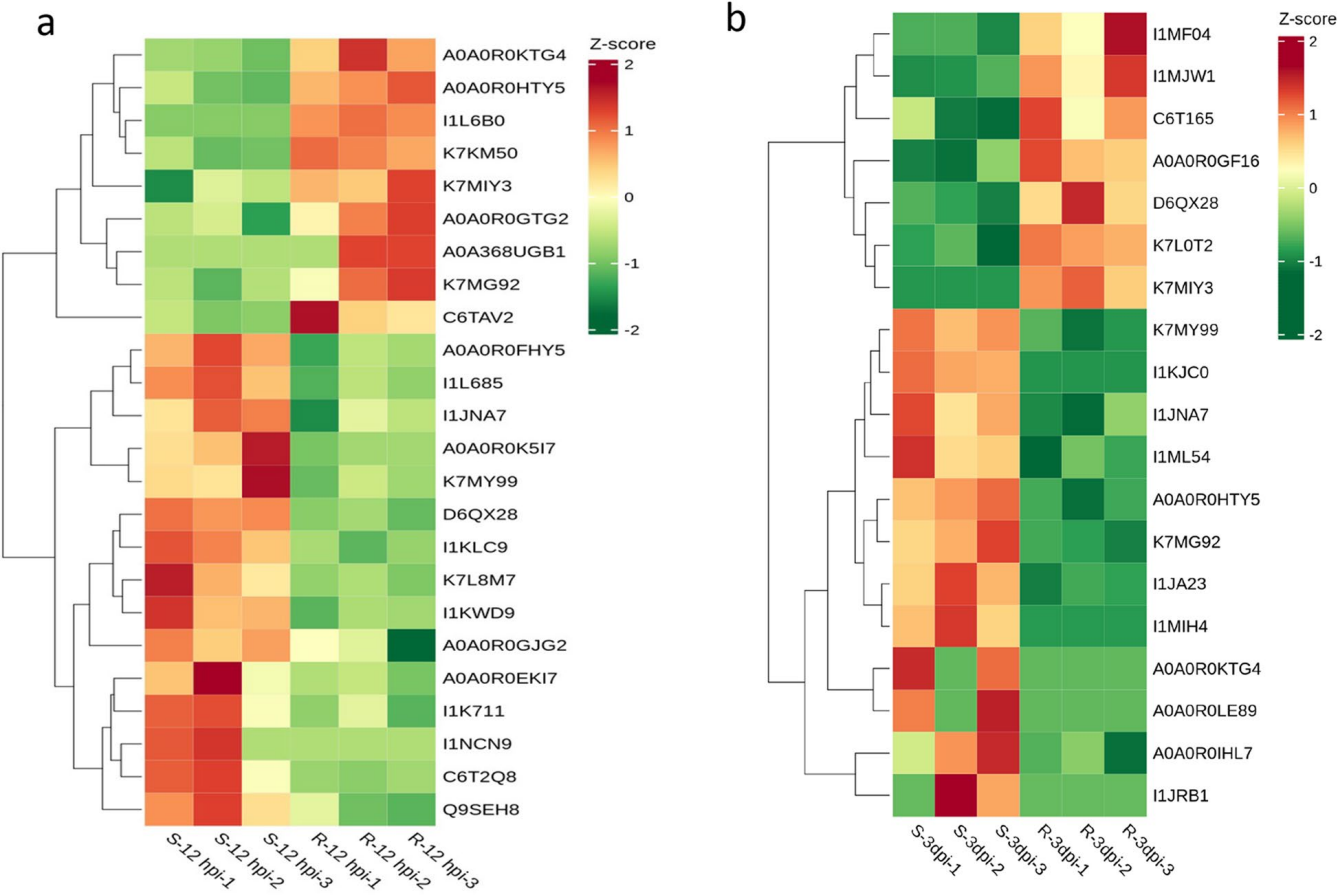
Heatmap of differentially expressed proteins in Ca^2^⁺ signaling pathway between R and S. **a** 12 h post-inoculation (**b**) 3 days post-inoculation

Our proteomic analysis revealed 24 calcium signaling-related DEPs at 12 h post-inoculation (hpi) (7 upregulated and 12 downregulated in resistant genotypes), decreasing to 19 DEPs by 72 hpi (Fig. 10a). Notably, only seven proteins (including CML30, CALM, and Annexin) exhibited sustained differential expression across both timepoints. This suggests stage-specific regulatory functions in defense. Constitutively modulated proteins such as Annexin may serve as core signaling nodes coordinating early perception and late effector responses. Intriguingly, Annexin 11 displayed downregulation at 12 hpi but upregulation at 3 days post-inoculation (dpi) in resistant plants (Fig. 10b). This biphasic expression pattern aligns with findings by Zhao et al. (2021), who demonstrated that *AtANN8* negatively regulates RPW8.1-mediated powdery mildew resistance and cell death, establishing Annexins as conserved immune modulators. Furthermore, the TIR-domain protein K7MIY3 was completely absent in susceptible cultivars at 3 dpi but maintained constitutive expression in resistant lines, suggesting its essential role in disease resistance.

### DEPs involved in regulation of ROS

One of the earliest responses in PTI is the rapid production of ROS, which exhibits direct antimicrobial activity while simultaneously amplifying defense signaling cascades. During early infection stages, we identified two differentially expressed proteins (02G308600 and 11G216500) that were significantly downregulated in disease-resistant plant material. Both proteins are functionally associated with ROS metabolic regulation. 02G308600 encodes an ABC1 atypical kinase-like protein, which has been demonstrated to play critical roles in plant growth, development, and stress responses (Yang et al. 2012). The second protein, 11G216500, represents a DELLA protein—a class of growth repressors known to be degraded through the ubiquitin–proteasome pathway in response to gibberellic acid (GA) signaling (Sun and Gubler 2004). DELLA proteins serve as integrative hubs for multiple hormone signaling pathways (Grant and Jones 2009) and have been shown to increase susceptibility to bacterial pathogens by suppressing SA-mediated defenses in *Arabidopsis* (Navarro et al. 2008). Notably, viral pathogens can exploit plant hormone pathways (Pallas and García, 2011), suggesting that the observed downregulation of these proteins may represent a strategic adaptation to maintain cellular homeostasis by preventing excessive ROS accumulation and mitigating oxidative stress.

### DEPs involved in MAPK cascades

The MAPK signaling pathway serves as a central hub in plant immunity, relaying signals from cell surface receptors to downstream defense mechanisms. During PTI, MAPK cascades are rapidly activated following PRR-mediated detection of PAMPs (Sun and Zhang 2022). Our investigation revealed six proteins that were upregulated in resistant plant material during early infection stages, three of which exhibited exclusive expression in disease-resistant genotypes: A cysteine-rich receptor-like protein kinase (CRK; A0A0R0EAZ5); A leucine-rich repeat transmembrane protein kinase (LRR-RLK; A0A0R0H9A9); A cysteine-rich secretory protein (CRISP; I1MNJ7). CRKs function as cell surface receptors that recognize PAMPs through their extracellular cysteine-rich domains, subsequently activating downstream defense pathways that trigger ROS bursts and defense-related gene expression (Zhang et al. 2023). The specific upregulation of CRK in resistant material underscores its potential pivotal role in early pathogen recognition and suggests it may be a crucial component of plant innate immunity. This is supported by studies demonstrating that TaCRK2 is essential for wheat defense against Puccinia triticina infection and positively regulates HR-associated cell death (Gu et al. 2020). Similarly, Saintenac et al. (2021) identified Stb16q as a plasma membrane-localized CRK conferring resistance to Zymoseptoria tritici by arresting early infection (Saintenac et al. 2021). LRR-RLKs utilize their extracellular leucine-rich repeat domains for specific ligand binding, while their intracellular kinase domains transduce signals to modulate defense-related hormone pathways (Li et al. 2002). The exclusive expression of LRR-RLK in resistant varieties indicates enhanced pathogen detection capacity, potentially leading to more robust immune activation. For instance, TaXa21, an LRR-RLK interacting with TaWRKY76 and TaWRKY62, positively regulates high-temperature seedling-plant resistance against Puccinia striiformis f.sp. tritici (Wang et al. 2019).

During later infection phases, we observed specific upregulation of the WRKY transcription factor I1KGT3 in resistant lines. The pivotal regulatory role of WRKY transcription factors in plant defense responses has been extensively documented (Birkenbihl et al. 2018; Liu et al. 2017; Luan et al. 2019; Pandey and Somssich 2009), where they orchestrate comprehensive transcriptional reprogramming of defense-related genes.

### DEPs involved in hormonal signaling pathways

SA serves as a pivotal phytohormone that governs plant immunity against biotrophic and hemibiotrophic pathogens. It orchestrates basal defense responses, amplifies localized immune reactions, and induces SAR through the transcriptional activation of pathogenesis-related (PR) genes (Peng et al. 2021). Our proteomic profiling of disease-resistant cultivars identified two critical regulatory nodes in SA signaling: the PR1 protein (I1ME54) and TGA-family transcription factors (A0A0R0GZ65). Notably, both components exhibited significant downregulation in resistant genotypes, which correlated with suppressed SA pathway activity. This finding suggests an evolutionary adaptation wherein resistant plants may strategically reallocate resources from constitutive SA-mediated defenses toward more specialized resistance mechanisms, potentially optimizing pathogen-specific responses over broad-spectrum immunity.

JA signaling pathway plays a central role in modulating plant–microbe interactions, maintaining a delicate balance between defense activation and symbiotic associations such as mycorrhization (Kadam and Barvkar 2024). Our comparative analysis revealed distinct JA pathway reprogramming in resistant materials: 1) Two COI1 isoforms showed reduced expression in resistant genotypes, indicating potential attenuation of canonical JA perception. This modulation may prevent excessive JA signaling that could lead to detrimental growth-defense trade-offs (Gimenez-Ibanez et al. 2015). 2) Resistant plants uniquely expressed two MYC-family transcription factors, suggesting isoform-specific recruitment for defense-related transcriptional reprogramming. These findings collectively highlight the sophisticated hormonal regulatory mechanisms that may underlie plant pathogen resistance.

### DEPs involved in secondary metabolism

Secondary metabolites have long been hypothesized to play a dynamic role in plant-pathogen interactions, representing one of the most extensively studied components of plant immune responses (Zaynab et al. 2018). Analogous to other evolutionarily conserved defense strategies, the biosynthesis and activation of these specialized metabolites are triggered by microbial detection systems—either through defense-related proteins or via the recognition of microbe-associated molecular patterns (MAMPs) by PRRs (Ahuja et al. 2012). Among these metabolites, dirigent proteins (DIRs) are particularly noteworthy, as they facilitate the biosynthesis of lignans and lignin-like compounds, which contribute significantly to plant defense mechanisms, secondary metabolism, and pathogen resistance (Li et al. 2017). Intriguingly, DIR genes exhibit pronounced upregulation in response to fungal and insect attacks, underscoring their critical role in plant immunity.

Our investigation into the molecular basis of rust resistance revealed distinct expression patterns of DIR proteins between resistant and susceptible genotypes following fungal infection. Specifically, seven DIR proteins were differentially expressed, with four upregulated and three downregulated in resistant plants. Notably, two DIR isoforms (A0A0R0LGP0 and I1JFU5) demonstrated resistance-specific induction, suggesting their potential functional specialization in defense against fungal pathogens.

### DEPs involved in fungal effector-triggered responses

Fungal pathogens employ sophisticated virulence strategies through the secretion of effector molecules that subvert host cellular processes and suppress plant immune defenses. In reciprocal evolutionary adaptation, plants have developed surveillance mechanisms to recognize these pathogen-derived effectors and mount effective defense responses. Among these, NLR receptors encoded by resistance (R) genes constitute a primary layer of intracellular immunity (Monteiro and Nishimura 2018). NLR proteins function as sophisticated immune sensors capable of detecting fungal effectors through both direct ligand binding and indirect recognition of effector-mediated host protein modifications. Upon activation, these receptors initiate robust immune signaling cascades that frequently culminate in the HR—a localized programmed cell death event that restricts pathogen proliferation at infection sites (Li et al. 2024). Our comparative transcriptomic analysis of NLR protein expression profiles between rust-resistant and susceptible cultivars revealed temporally dynamic patterns:Early infection phase (12 hpi): Identification of twelve DEPs with balanced regulation (6 up-/6 down-regulated); Later infection stage (3 dpi): Shift to fourteen DEPs showing predominant suppression (4 up-/10 down-regulated); Resistance-specific expression: Five NLR proteins (A0A0R0KET2, I1KI39, I1M0N5, K7K1J3, I1MBG6) exhibited genotype-specific expression exclusively in resistant plants.

These temporal expression patterns suggest an initial balanced immune activation during early infection that becomes progressively compromised in susceptible plants, as evidenced by the predominant downregulation observed at 3 dpi. The resistance-specific NLR proteins likely play crucial roles in pathogen recognition or downstream defense signaling pathways. Future studies should focus on: functional validation of these candidate NLRs through reverse genetic approaches,elucidation of their potential interactions with corresponding fungal effectors, and characterization of their signaling networks. Such investigations will provide critical insights into the molecular mechanisms underlying rust resistance in this pathosystem.

### Dynamic cell wall remodeling in plant defense

Plant-pathogen interactions trigger extensive spatial reorganization of defense-related proteins to reinforce both physical and chemical barriers against microbial invasion. Among these defensive strategies, cell wall remodeling represents a critical frontline defense mechanism involving the coordinated action of specialized enzymes including peroxidases (PODs), callose synthases, and xyloglucan endotransglucosylases/hydrolases (XTHs) (Bacete et al. 2018; Planas 2022). This dynamic restructuring process enhances the structural integrity and biochemical composition of the cell wall to impede pathogen progression.

The plant peroxidase system plays a dual role in pathogen defense through structural reinforcement by catalyzing oxidative cross-linking of phenolic compounds (lignin, suberin) to strengthen the cell wall matrix, and through ROS modulation by fine-tuning redox homeostasis during immune responses. Our comparative proteomic analysis identified 19 differentially expressed PODs in rust-resistant genotypes, revealing three distinct temporal expression patterns: early-phase responders (12 hpi) with 5 PODs (2 up-/3 down-regulated), late-phase responders (3 dpi) with 9 PODs (4 up-/5 down-regulated), and sustained responders with 5 PODs maintaining differential expression across both phases (2 up-/3 down-regulated). Notably, the consistently up-regulated PODs (particularly I1MBG6 and A0A0R0KET2) demonstrate strong correlation with resistance phenotypes. These findings are supported by functional validation studies showing that TaGPX3.2A silencing via virus-induced gene silencing (VIGS) significantly impaired wheat resistance to Fusarium graminearum (*P* < 0.01), establishing peroxidase-mediated cross-linking as a biochemical determinant of disease resistance (Jiang et al. 2023).

Xyloglucan endotransglucosylase/hydrolase (XET) enzymes orchestrate hemicellulose matrix reorganization through cleavage and re-ligation of xyloglucan polymers, modulation of cell wall extensibility and tensile strength, and regulation of apoplastic defense compound deposition (Li et al. 2021; Miedes et al. 2011). Our proteomic profiling identified five XTH family members showing significant upregulation (> twofold change, FDR < 0.05) in resistant cultivars, including the novel XET isoform I1LH32 exhibiting genotype-specific expression, K7K1J3 showing sustained induction throughout infection, and I1M0N5 as a late-phase responder with peak expression at 3 dpi. Functional characterization suggests these XET isoforms may contribute to resistance through three synergistic mechanisms: enhanced apoplastic barrier formation via xyloglucan cross-linking, dynamic wall loosening facilitating defense compound transport, and maintenance of wall plasticity under pathogen-induced stress.

The identification of these differentially expressed cell wall-modifying enzymes provides valuable molecular markers for resistance breeding programs, potential targets for genetic engineering of durable resistance, and new insights into spatiotemporal regulation of wall remodeling during defense. These findings significantly advance our understanding of the sophisticated mechanisms plants employ to reinforce their cellular barriers against pathogen invasion.

### Organelle-specific defense responses in plant immunity

Plants have evolved sophisticated organelle-specific defense mechanisms to combat pathogen attacks. Upon pathogen recognition, a series of signaling events orchestrate the nuclear translocation of key defense-related transcription factors including WRKY, MYB, and TGA family members. These translocated transcription factors then activate the expression of crucial defense genes encoding pathogenesis-related proteins and phytoalexins, forming the molecular basis of plant immunity (Wu et al. 2022). Among these, the MYB transcription factor superfamily represents one of the most functionally diverse groups in plants, playing regulatory roles in diverse biological processes ranging from developmental programming and secondary metabolite biosynthesis to environmental stress responses.

Our comparative transcriptomic analysis revealed 11 differentially expressed transcription factors between resistant and susceptible plant materials, comprising 6 upregulated factors that potentially function as positive regulators of resistance and 5 downregulated factors that may act as negative regulators or pathogen-suppressed components. These transcription factors were distributed across three major families: six MYB-type factors likely involved in phenylpropanoid metabolism and cell wall reinforcement, four WRKY-type factors potentially mediating salicylic acid and jasmonic acid signaling pathways, and one TGA2 factor (a bZIP family member) that may cooperate with NPR1 to activate systemic acquired resistance.

Particularly noteworthy was the identification of four proteins (A0A0R0HT22, K7MER4, K7N0C2, and I1KGT3) showing exclusive expression in resistant varieties, suggesting their potential utility as molecular markers for disease resistance. However, comprehensive functional validation through gene silencing, overexpression studies, and protein interaction analyses will be essential to confirm their specific roles in plant defense mechanisms.

These findings significantly advance our understanding of the spatial organization of plant immune responses and provide valuable molecular targets for crop improvement. Future research directions should focus on elucidating the precise regulatory networks governing these organelle-specific responses and translating this knowledge into practical applications for developing disease-resistant crop varieties through molecular breeding approaches. The integration of fold-change quantification and statistical validation of these transcriptional regulators would further strengthen the biological significance of these observations.

### Proteome-level persistence of defense mechanisms unveils the molecular landscape of soybean rust resistance

Our 4D-DIA proteomic analysis both corroborates and significantly extends previous transcriptomic and metabolomic findings on soybean rust resistance. While earlier transcriptomic studies by Panthee et al. (2007, 2009) and Soria-Guerra et al. (2010) identified growth-stage-specific upregulation of SA-related genes, GST, peroxidases, and phenylpropanoid pathway components, our study reveals that resistant genotypes exhibit sustained protein-level activation of these defense mechanisms, which are often transient or absent in susceptible plants.

We confirm the crucial role of conserved pathways such as phenylpropanoid/isollavonoid biosynthesis and ROS homeostasis, consistent with findings by Hossain et al. (2018) and Silva et al. (2021). However, our proteomic approach provides novel insights into the spatiotemporal regulation of defense responses, including: precise modulation of SA/JA signaling; organelle-specific defense strategies in chloroplasts and membranes; biphasic expression of calcium sensors like Annexin 11; and significant upregulation of DIR proteins and XTHs involved in cell wall remodeling. Furthermore, WGCNA identified resistance-specific co-expression modules enriched in NLRs, MAPK components, and ER stress responders, corroborating genomic studies by Ratnaparkhe et al. (2020) while adding protein-level validation.

Notably, we observed suppression of photosynthesis-related proteins and upregulation of chlorophyll catabolism-associated proteins in susceptible plants, aligning with Tremblay et al.'s (2013) emphasis on the pathogen's dependence on host energy metabolism and nonhost resistance mechanisms in Medicago (Ishiga et al. 2015). Unlike the transient defense activation reported in earlier transcriptomic studies, our proteomic data demonstrate sustained defense induction in resistant plants and progressive suppression in susceptible ones, highlighting the potential importance of protein-level stability and post-translational regulation in rust resistance.

These findings reinforce the value of pyramiding multiple resistance mechanisms—including NLR genes, cell wall modifiers, and secondary metabolite pathways—as proposed by Langenbach et al. (2016). Additionally, the identification of highly conserved effector targets supports their use in RNAi-based control strategies (Ouyang et al. 2024), offering a complementary approach to genetic resistance.

## Research questions and future prospects

This study highlights the dynamic interplay between PTI and ETI in plant defense against rust infection. Key findings include the spatiotemporal regulation of calcium signaling, ROS homeostasis, and NLR-mediated resistance, alongside hormonal trade-offs (SA vs. JA) and cell wall remodeling via DIRs and XETs. However, critical questions remain, such as how specific calcium sensors (e.g., Annexin 11) modulate immune transitions, why NLRs are suppressed in susceptible plants, and whether DIR-mediated lignification directly inhibits fungal growth.

Future research should prioritize functional validation of resistance-linked genes (e.g., CRK, NLRs, I1LH32) using CRISPR editing and effector screening, alongside high-resolution spatiotemporal analyses (single-cell transcriptomics, live imaging) to dissect early defense signaling. Translational applications include marker-assisted breeding for stacked resistance genes and chemical priming to enhance immunity. Additionally, exploring evolutionary pathogen-host dynamics and microbiome interactions could reveal novel resistance mechanisms.

By addressing these gaps, this work paves the way for durable rust-resistant crops, reducing reliance on fungicides. Integrating multi-omics, genome engineering, and field trials will bridge mechanistic insights with real-world crop protection, offering sustainable solutions for global food security.

## Conclusion

Based on 4D-DIA proteomic analysis, this study systematically elucidates the protein-level molecular basis of soybean rust resistance, demonstrating that resistant cultivars deploy a multi-layered defense system through sustained upregulation of immune receptors (CRKs, LRR-RLKs), MAPK signaling components, and cell wall remodeling proteins (peroxidases, XTHs), along with dynamic modulation of calcium signaling and ROS homeostasis, and activation of phenylpropanoid/isoflavonoid biosynthesis and ER stress response pathways. The study identifies chloroplasts and membranes as key defense hubs, and reveals a set of resistance-associated proteins including DIR proteins, XTHs, and NLRs. Unlike susceptible plants, resistant genotypes exhibit persistent activation of defense responses at the protein level rather than transient induction. These findings not only provide a protein-level blueprint of soybean rust resistance mechanisms but also offer crucial candidate genes and a theoretical foundation for marker-assisted breeding and genetic engineering to develop durable rust-resistant soybean varieties, contributing significantly to reducing reliance on fungicides and achieving sustainable soybean production.

## Materials and methods

### Protein extraction

Protein extraction was performed using an acetone precipitation method with the following modifications. Briefly, frozen leaf tissues were cryogenically pulverized in liquid nitrogen using a pre-chilled mortar and pestle. The powdered samples were then homogenized in ice-cold L3 extraction buffer (1% SDS, 100 mM Tris–HCl pH 8.0, 7 M urea, 2 M thiourea, 1 mM PMSF, 2 mM EDTA) at a 1:5 (w/v) tissue-to-buffer ratio. The homogenate was vortexed vigorously for 1 min, followed by intermittent sonication (5 s pulses with 10 s intervals) on ice for 10 min to ensure complete cell lysis. Cellular debris was removed by centrifugation at 12,000 × g for 15 min at 4 °C. For protein precipitation, a four-fold volume of pre-chilled (−20 °C) acetone was added to the clarified supernatant and mixed gently by inversion. The mixture was precipitated overnight at −20 °C to maximize protein recovery. The precipitated proteins were collected by centrifugation at 10,000 × g for 10 min at 4 °C, followed by two washes with ice-cold 80% acetone to remove residual SDS and salts. The final protein pellet was air-dried for 5 min and solubilized in 8 M urea buffer with gentle vortexing. Protein concentration was determined in triplicate using a Pierce™ BCA protein assay kit (Thermo Fisher Scientific) according to the manufacturer's instructions, with bovine serum albumin as the standard.

### Digestion and cleanup

Equal protein amounts from each sample were subjected to tryptic digestion. Proteins were solubilized in 200 μL of 8 M urea, reduced with 10 mM DTT (37 °C, 45 min), and alkylated with 50 mM iodoacetamide (IAM, room temperature, 15 min in darkness). Proteins were then precipitated with 4 × volume of chilled acetone (− 20 °C, 2 h), centrifuged, and air-dried. The pellet was resuspended in 200μL of 25 mM ammonium bicarbonate and digested overnight at 37 °C with 3 μL trypsin (Promega). The resulting peptides were desalted using a C18 cartridge, vacuum-dried, and reconstituted in 0.1% (v/v) formic acid.

### LC–MS/MS analysis

Peptide separation was performed using a nanoElute UHPLC system (Bruker Daltonics) with a 40-min gradient at 0.3 μL/min flow rate. Approximately 200 ng of peptides were loaded onto a reverse-phase C18 column (Aurora Series, 25 cm × 75 μm ID, 1.6 μm, IonOpticks) equipped with an integrated CaptiveSpray Emitter, maintained at 50 °C by a column oven. The mobile phases consisted of: Phase A: 0.1% formic acid in water; Phase B: 0.1% formic acid in acetonitrile. The gradient profile was as follows:2–22% B (0–25 min), 22–35% B (25–30 min), 35–80% B (30–35 min), 80% B isocratic (35–40 min).

Mass spectrometry analysis was conducted on a timsTOF Pro2 (Bruker Daltonics) equipped with a CaptiveSpray nano-electrospray ion source (1500 V capillary voltage). For diaPASEF method development, the instrument was operated in PASEF mode with:4 PASEF MS/MS frames per complete frame, m/z range: 100–1700, Ion mobility range: 0.85–1.3 Vs/cm^2^, Target value: 10,000 (intensity threshold: 1500), Collision energy gradient: 27–45 eV (1/K0 = 0.85–1.3 Vs/cm^2^), Dynamic quadrupole isolation: 2 Th (m/z < 700) or 3 Th (m/z > 800).

In diaPASEF mode, the instrument control software was extended to define quadrupole isolation windows as a function of the TIMS scan time. Seamless and synchronous ramping of all applied voltage is achieved by modifying the instrument control electronics. We defined 25 Th isolation windows from m/z about 400 to 1200 and totally 48windows were defined. Other parameters were the same as DDA-PASEF mode.

### Database search and quantification

MS raw data were analyzed using DIA-NN (v1.8.1) with a library-free method. The Uniprot-proteome_UP000008827-20221006_dadou.fasta database (a total of 74,863 sequences) was used to create a spectral library with deep learning algorithms of neural networks. The option of MBR was employed to create a spectral library from DIA data, and then the data were reanalyzed using this library. The FDR of search results was adjusted to < 1% at both the protein and precursor ion levels; the remaining identifications were used for further quantification analysis.

### Bioinformatics analysis

For DEPs in each comparison group, we conducted enrichment analyses across four dimensions: GO terms, KOG categories, KEGG pathways, and protein domains. The enrichment significance (P-value) was calculated using the hypergeometric distribution algorithm to determine whether DEPs showed statistically significant enrichment in specific functional categories compared to the background proteome (all identified proteins). Additionally, subcellular localization and signal peptide predictions were performed to gain more comprehensive insights into the physiological functions associated with the DEPs.

### Quantitative real-time PCR analysis

Total RNA was extracted from soybean leaves using TRIzol reagent (Invitrogen) and treated with DNase I to remove genomic DNA, followed by cDNA synthesis using HiScript® II Q RT SuperMix (+ gDNA wiper) kit (Vazyme). qRT-PCR was performed in triplicate for each sample on a CFX Connect Real-Time System (Bio-Rad) using SYBR Green, with cycling conditions of 45 cycles at 95 °C for 10 s and 60 °C for 30 s, and fluorescence detection at each cycle end. β-actin served as the internal reference gene for normalization, and relative gene expression levels were calculated using the 2^−ΔΔCt^ method, with three biological replicates (each pooled from ≥ 5 individual plants) analyzed per tissue type.

### WPCNA analysis

WPCNA algorithm is a widely used systems biology approach that constructs gene co-expression networks from high-throughput mRNA expression data by first assuming a scale-free network distribution, then calculating gene co-expression correlation matrices and adjacency functions to generate hierarchical clustering trees where distinct branches represent functionally coherent gene modules with high intra-module and low inter-module co-expression connectivity, ultimately enabling the identification of phenotype- or disease-associated gene networks and potential therapeutic targets through module-trait relationship analysis.

WPCNA analysis constructs protein co-expression networks by building cluster dendrograms based on expression correlation patterns, where each color-coded module represents a group of proteins with similar expression profiles across different physiological conditions or tissues, suggesting potential functional relationships, with the vertical distance in the upper dendrogram portion indicating the degree of dissimilarity between protein nodes (shorter distances denoting stronger co-expression) while horizontal spacing is merely for visual arrangement without quantitative meaning.

## Supplementary Information

Supplementary Material 1: Table S1 GO.enrichment of rust pathogen response in resistant and susceptible materials.

Supplementary Material 2: Table S2 Enriched proteins in the purple module.

Supplementary Material 3: Table S3 Enriched proteins in the tan module.

Supplementary Material 4: Table S4 Primers List for q-PCR.

## Data Availability

The data that support the findings of this study are available from the corresponding author upon reasonable request.

## Abbreviations

DEP: Differentially expressed protein
ETI: Effector-triggered immunity
PTI: Pattern-triggered immunity
PRRs: Pattern recognition receptors
PAMPs: Pathogen-associated molecular patterns
RLKs: Receptor-like kinases
RLPs: Receptor-like proteins
ROS: Reactive oxygen species
MAPK: Mitogen-activated protein kinase
R: Resistance
NLR: Nucleotide-binding leucine-rich repeat
WPCNA: Weighted Protein Co-expression Network Analysis
